# Retraction: SRCIN1 Suppressed Osteosarcoma Cell Proliferation and Invasion

**DOI:** 10.1371/journal.pone.0282984

**Published:** 2023-03-29

**Authors:** The PLOS ONE Editors

The *PLOS ONE* Editors retract this article [1] because it was identified as one of a series of submissions for which we have concerns about peer review, authorship, and similarities across articles. Image similarities were noted between Fig 1A in [1] and Fig 1A in [2,3], between Fig 3E in [1] and Fig 3B in [4] and Fig 6A in [5], and between Fig 5A in [1] and Fig 2D in [6,7]. These concerns call into question the validity and provenance of the reported results. We regret that the issues were not addressed prior to the article’s publication.

All authors either did not respond directly or could not be reached.
